# Ibero-American nursing context: constructing and sharing knowledge

**DOI:** 10.1590/0034-7167.2024770101

**Published:** 2023-12-08

**Authors:** José Ramón Martínez-Riera

**Affiliations:** I Universidad de Alicante, Professor Titular Departamento de Enfermagem Comunitária, Medicina Preventiva e Saúde Pública e História da Ciência. Alicante, Spain

*To Community Nursing Association (AEC) and Latin American Association of Schools and Colleges of Nursing (ALADEFE), for their contributions to the construction of nursing knowledge.*

*“La realidad será lo que seamos capaces de construir”*

“Dispara, yo ya estoy muerto” (2013), Julia Navarro*

Next week, two major scientific events will take place in Madrid and Granada (Spain). These will be international, specific activities in a very special way, although not exclusive to the Ibero-American field, organized and developed by two very important scientific nursing societies, such as Community Nursing Association (AEC) and *Latin American Association of Schools and Colleges of Nursing (ALADEFE)*, with international implementation and influence.

The AEC, with almost 35 years of evolution, celebrates what will be its VII International Congress with the motto *“Vulnerability and Community Health: a new era for determinants of health”* as part of its principles with the health of the community, families and people it cares for. On this occasion, it focuses special attention on responding to vulnerable populations’ care needs, while also analyzing and reflecting on the important influence exerted by determinants of health. Without going into detail about the different aspects that will be addressed at the tables, workshops and colloquiums that make up the Congress, it is worth to mention the importance of carrying out a comprehensive, integrated and integrative approach to health such as the one carried out and which involves a perspective of nursing that, furthermore, is shared with other disciplinary perspectives to try to identify the needs that these communities have, but also to propose what can or should be the responses that, as nurses, we give them in a context that transcends the local or the national, to extend globally to the Ibero-American sphere.

ALADEFE, in turn, with a similar history to that of AEC, 37 years old, proposes as the theme of its XVII Ibero-American Nursing Education Conference: *“Research in Nursing Education: constructing an Ibero-American context for caring for people”*. In this case, nurses raise the need to debate what and how the training of future nurses should be, in a global perspective that also allows the construction of an Ibero-American nursing context that meets the care needs of individuals, families and the community from a transcultural, transdisciplinarity and transsectoral perspective that facilitates people-centered approaches.

In relation to the temporal (in the week of October 23 to 28) and spatial (Spain) coincidence, it is considered essential to combine the complementarity that both scientific approaches make, a reality that requires rigorous, close, effective and efficient responses, such as those that Ibero-American nurses, committed to their profession, but in a very special way to society and their right to dignified and accessible health care, are willing and able to propose and contribute.

The configuration of a map of conferences, congresses, conferences, symposia must be seen and assumed as an opportunity and a force in the construction of knowledge, sharing experiences, perceptions, evidence, and not just another way of doing tourism, as happens with other aspects such as gastronomic, cultural, oenological. Scientific events must constitute a considered and transcendent decision by those who organize them, identifying and raising current issues of professional, disciplinary and scientific interest that, at each moment, are more relevant and priority, carried out with rigor and innovation, as well as those who decide to opt for one or the other to reinforce their professional scientific development, not only with their assistance, but also with the commitment to carry out an active and real participation in their scope.

Our contribution to the health of individuals, families and the community goes far beyond the systematic, standardized or routine provision of care, the application of diagnostic, therapeutic or exploratory techniques, the management of resources or care or nursing education. As nurses and, therefore, professionals of a science and a discipline, regardless of the area in which we act as such, we have a moral, ethical and aesthetic obligation to do so with the strictest scientific rigor that guarantees the provision of care, teaching or management with the quality that they deserve and should be offered to the people with whom we interact, whether in health or illness, in a health center or hospital, at school or work, at home or in the community where they are integrated to promote or maintain their health, prevent or care for their illness, promote their recovery and integration or follow them in mourning.

This means that we have to acquire the commitment to engage in our permanent, ongoing and lifelong training. Thinking that they can work as a nurse simply because they have a diploma that certifies them as such is a huge mistake and an unacceptable and irresponsible attitude. A university degree officially qualifies them to work as a nurse, but being and feeling like a nurse, with everything that means, goes far beyond having the legal capacity to do so. It represents a professional, scientific, social and human responsibility that we must assume and, therefore, always remain active.

Based on what has been said, participation in professional scientific activities must be identified as a fundamental part of our development. Learning, absorbing and sharing knowledge is inherent to being, feeling and acting as a nurse. Failure to do so is a fraud on the profession and society, which trusts in our attitude and ability to provide quality professional care.

In this regard, it is paradoxical to propose, as is being done, the need to increase the number of courses that constitute the nursing course, or the number of years of training in the specialties. Because this approach comes up against the unfortunate fact that, regardless of the duration of studies or specialized training, we are unable to identify, respect and promote the experience and skills acquired, doing so only based on seniority in the position and ignoring individual involvement and commitment to always remain updated, dynamic and adapted to scientific-professional and social evolution. Continuing to maintain the falsely supportive belief and attitude that a nurse has the same responsiveness, knowledge or rigor, or the same ability to deal with the complex health problems they face on a daily basis, regardless of being able to prove this ability or knowledge, just by virtue of being a nurse, it leads to a collective deterioration of our development and subsequent academic, professional or scientific recognition. It is not about establishing hierarchies, but about trying to justify that we are all equal based on the fact that we have a degree or the number of years it takes to obtain it. It is essentially an absolute lack of professional and scientific maturity that requires an immediate change of attitude that allows the foundations of nursing to be laid. If we want, of course, to have the respect and consideration from the scientific and professional community and society as a whole, we must seriously consider maturing and reaching the level that corresponds to us as a science and discipline. The opposite inevitably leads us to mediocrity and inconsequence which, logically, are not combatted with the fruitless cry of victimistic lament^(1-2)^.

Therefore, and beyond the temporal considerations of the duration of studies or specialized training, what we must bear in mind is the imperative need to analyze the contents that make up current study plans to identify whether they really respond to current care needs and demands or, on the contrary, comply with opportunity criteria related to the demands of health systems which are the main employers of nurses. We cannot continue to allow the training of future nurses to be hijacked by interests that move away from the true objective, which, as nurses, we must meet. In other words, the social dynamics that regulate the search for care based on health determinants, health assets or sustainable development goals (SDGs) must be addressed, which must be identified and valued in the dimension they represent and in which, without a doubt, nurses have the obligation to make a unique, specific and autonomous contribution based on transdisciplinary work. Failure to do so contributes to feeding and perpetuating the current medicalized model based on social welfare and fragmented and assuming a subsidiary role in which care is relegated to the domestic sphere where it is neither visible, nor recognizable, nor valued, not meeting the population’s needs.

All of this means that the coincidence of these two scientific activities to be carried out next week acquires a meaning that goes beyond their celebration, being a fundamental reference that not only gives them great potential for building knowledge, but also complements their approaches, objectives and future challenges to share them and be able to undertake this necessary construction of the Ibero-American context of nursing.

Although it is true that the overlapping of dates makes it impossible, in this case, to achieve the desirable ubiquity that would allow us to be in both spaces at the same time, it is no less true that the conclusions reached in both events must be shared to enrich the final contribution of AEC and ALADEFE.

The construction of nursing knowledge must contribute to the necessary maturation of nurses - and not nursing, although logically it is fed by their contributions -, sharing it, as it is not the exclusive property of anyone. Only through generosity in sharing knowledge can we build a context as desirable and necessary as the context of Ibero-American nursing. From training to direct care, through management and research that enable the visibility and appreciation of professional nursing care as a way of sustaining our scientific-professional reference and achieving the respect they deserve with the offer of quality, affection and humanity that we nurses offer.

AEC and ALADEFE offer us a favorable scenario for building the Ibero-American nursing context. Now it is up to nurses to know how to take advantage of it to exercise transformative leadership that assumes the necessary responsibility, being creative, innovative, proactive, assertive, risk-taking, optimistic and fighting for a better society. Nursing contribution to professional care must be valued; train nurses for the community; identify and value an area of specific and autonomous competence that takes into account aspects of caring in, with and for the community, taking responsibility for our skills; generate healthy spaces, supporting people, families and the community to achieve their empowerment in a shared process of creating health; articulate community resources, identifying felt care needs; promote autonomy and self-care; focus attention on people and their health, promoting active community participation in the development of life skills; promote changes in healthy behaviors and habits; and transform the health system into a salutogenic perspective, respecting transculturality, favoring transsectorality and working transdisciplinary^(3)^.

The identification of differences in the environments that make up the Ibero-American nursing context must be seen as an opportunity for enrichment and never as an obstacle to its construction.

In turn, AEC and ALADEFE, together with other scientific societies, organizations or professional representatives, must promote spaces for analysis, reflection and collective construction of nursing knowledge to share it and enable progress which allows us to act with decision, firmness and security, supported by our own knowledge, which must necessarily be shared with those from other disciplines and with those in society itself, who must participate in making decisions about their health.

Congratulations to AEC and ALADEFE, for their work, effort and faith in what should be an international reference in professional care, and to the IBERO-AMERICAN NURSING CONTEXT.

## Footnotes

* Spanish journalist and writer (1953).
